# Understanding the impact of local funding cuts on environmental health and regulatory services and gastrointestinal infection outcomes: a longitudinal ecological study protocol

**DOI:** 10.3310/nihropenres.13426.1

**Published:** 2023-08-14

**Authors:** Lauren Murrell, Helen Clough, Roger Gibb, Xingna Zhang, Mark Green, Marie Chattaway, Iain Buchan, Benjamin Barr, Daniel Hungerford

**Affiliations:** 1 Health Protection Research Unit in Gastrointestinal Infections, University of Liverpool, National Institute for Health and Care Research, Liverpool, England, L69 7BE, UK; 2 Department of Clinical Infection, Microbiology and Immunology, University of Liverpool, Institute of Infection, Veterinary and Ecological Sciences, Liverpool, England, UK; 3 Department of Public Health, Policy & Systems, University of Liverpool, Institute of Population Health, Liverpool, L69 3GF, UK; 4 Department of Geography and Planning, School of Environmental Sciences, University of Liverpool, Liverpool, L69 3GF, UK; 5 United Kingdom Health Security Agency (UKHSA), London, NW9 5EQ, UK

**Keywords:** Gastrointestinal infection, local funding cuts, environmental health and regulatory services, inequalities, Food safety, Food hygiene

## Abstract

**Background:**

Gastrointestinal (GI) infections result in 17 million cases annually, with foodborne illness costing the National Health Service (NHS) £60m per year. The burden of GI infection is unequally distributed, with greater impact in more socioeconomically disadvantaged groups and areas. Local authorities (LA) provide vital services that protect public health and wellbeing. The impact of funding cuts to local services and their effect on public health is an area of concern. Environmental health and regulatory (EH) services are responsible for roles such as food safety and infectious disease control. This study aims to understand the impact of local funding cuts on EH and GI infection outcomes.

**Methods:**

We will conduct an ecological longitudinal study in England from 2010-2019 at the LA level to examine how changes in EH expenditure overtime have impacted EH and GI infection outcomes. Data will be gathered on food hygiene enforcement, food hygiene compliance levels, GI infection hospitalisation, NHS 111 calls relating to GI infection symptoms, GI infection pathogen data, deprivation, and population density. Measures will be aggregated to LA level and statistical analysis will be carried out.

**Ethics and dissemination:**

University of Liverpool Ethics committee have confirmed ethical approval will not be required. All data will be aggregated and anonymised, therefore only data sharing agreements will be required. Findings will be disseminated to the stakeholder group in addition to outputs through conferences and publications. These findings will help understand impact of key services on public health and should inform government and public health policy and strategy.

## Plain language summary

Stomach bugs can be spread by unclean water, food, surfaces, and cause illness such as diarrhoea and vomiting. These illnesses have affected people from poorer backgrounds and of different ethnic backgrounds more. Local authorities in England run environmental health services which carry out jobs that are good for the health and safety of the people of that area. An important job is making sure places such as restaurants, bars, hotels, cafes, and canteens are safe to eat from. This is important, as a lot of people get sick each year from eating unclean food.

Since 2010, the amount of money given to these services has been cut. We want to know how these cuts have changed the way environmental health services are able to do their jobs, and to find out if these service changes are linked to the amount of stomach bugs reported. Using existing data for England and statistical methods we will discover if these changes are different by area, over time and if poorer areas are affected more than those less poor.

We will use information on local authorities in England from 2010 – 2019. We will use information on the amount of money each area gets, in addition to information on how services carry out jobs to make food and food places safe for eating. We will see if this is linked to changes in stomach bug sickness by looking at healthcare data on the number of people in hospital with stomach bugs, and the number of NHS calls made by people with vomiting and diarrhoea. We will also look at the type and number of bugs reported from laboratories.

This study will give the government important information to make funding and service decisions in the future and to protect public health.

## Introduction

Gastrointestinal (GI) infections in the UK cause substantial morbidity and mortality in addition to placing significant stress on health care services. There are estimated to be 17 million cases of infectious intestinal disease (IID) and 1 million GP consultations due to IID annually ^1^. In 2018-19 there were an estimated 119,000 emergency hospital admissions due to illness with GI infection. Transmission occurs by exposure to viral, bacterial or fungal pathogens through contaminated environment, water or food sources, and human or animal contact. Foodborne GI infections place a large strain on health systems, costing the NHS an estimated £60m annually ^2^. Foodborne transmission accounts for a substantial proportion of GI infection. Foodborne pathogens account for an estimated 2.4 million cases of disease in the UK annually, these pathogens include Campylobacter, Listeria STEC, Salmonella and norovirus. A large proportion of illness is acquired by eating out at food establishments and takeaways, for example this route is estimated to be responsible for 37% and 26% of foodborne norovirus cases, respectively ^3^.

Sociodemographic patterning of GI infection amongst the UK population is evident. Epidemiological studies report an unequal distribution in risk of GI infection and its outcomes amongst those of different ethnic backgrounds and of disadvantaged socioeconomic background ^4– 7^. A cross sectional analysis using UK population data from the IID2 survey identified increased symptom severity amongst those of more disadvantaged socioeconomic backgrounds across all age groups, in turn accounting for higher absence from school or work also reported amongst this group ^7^. Research suggests there are higher rates of hospital admissions amongst those from deprived socioeconomic backgrounds in the UK, evidenced in an ecological ^5^ and retrospective case control study ^6^. This is supported by a longitudinal ecological study in England that identified increased rates of hospitalisation in areas of higher unemployment - as a measure of deprivation, in addition to areas with higher percentage of people from a Pakistani background ^4^. There is also sociodemographic heterogeneity in the pathogen which causes GI infections in the UK. Varying by area and socioeconomic deprivation ^8– 11^ and ethnicity ^8– 12^. This heterogeneity is related to the interplay between sociodemographic factors, risk activities and transmission routes. Though sociodemographic and spatial inequalities of GI infection are well described, the drivers behind them remain to be understood.

Local authorities in the UK provide key services for the populations of these areas, including public protective services, vital for health protection and public wellbeing. Since the introduction of austerity in 2010, local authorities have been subject to a reduction in funding of almost 50%. The decline in resources available for the delivery of these public services and how this may in turn impact public health is an area of concern.

As a public protective service, the environmental health and regulatory (EH) services are the local authority body responsible for food safety and infectious disease control. The local authority is responsible for the delivery of food hygiene controls at food premises. These include roles such as surveillance, interventions, inspections and sampling and enforcement, mainly carried out by environmental health officers (EHO’s). However, the reduction in funding in recent years has impacted on local authorities’ ability to carry out statutory and regulatory functions.

A report by the National Audit Office based on local authorities in England estimated that between 2012/13 and 2017/18 there has been a reduction in spending on food hygiene controls by 19%. It also identified that some local authorities are not meeting statutory food hygiene objectives, including reduced environmental sampling. The local authorities contacted attributed the reductions in sampling to reduced food hygiene staffing levels, which have decreased by an estimated 13% between 2012/13 and 2017/18. Similar trends were identified in a report covering food safety in England and Wales where EHO numbers reduced by a third, in addition to substantial reductions in sampling, prosecutions and other enforcement actions over recent years.

Increased expenditure on food safety and sanitation services have shown to be associated with reductions in notifiable enteric disease ^13^. Reports indicate that funding cuts are placing significant pressures on EH services, and whilst the evidence base for the impacts of local funding cuts on public health is developing, a gap remains regarding the impact that cuts to EH may have on GI infection outcomes. To better understand this relationship, it is crucial to investigate the impacts of local funding cuts on EH services overtime and examine how these outcomes may be associated with GI infection outcomes. A longitudinal ecological approach will be used to examine the association of changes in EH expenditure on key indicators of EH in the context of food hygiene and investigate the association between these impacts and GI infection outcomes. We will explore how outcomes vary by area and by level of deprivation.

## Methods

### Patient and Public Involvement

This project will incorporate public and patient involvement and engagement (PPIE) throughout. To date, a PPIE panel has been held, in which preliminary plans were presented and variables were discussed. In put was used in deliberation of variables of analysis. The PPIE group will be involved in the development of the protocol in addition to steps following this throughout the remainder of the study. 

### Study aim and objectives

The aim of the study will be to understand the impact of changes to EH service expenditure on EH services indicators, and how this may impact GI infection.

The main objectives of the study are to:

1. Describe the changes in expenditure of EH services, and how they vary by local authority (LA) and sociodemographic factors between 2010/11 and 2018/19
2. Describe key EH indicators, and how they vary by LA and sociodemographic factors between 2010/11 and 2018/19
3. Examine how changes in expenditure in EH services are associated with trends in EH indicators overtime, and how this association may vary by LA and sociodemographic factors.
4. Describe GI infection outcomes by LA and socioeconomic and sociodemographic factors between 2010/11 and 2018/19
5. Investigate if changes in EH indicators are associated with GI infection outcomes and how this association may vary by LA and sociodemographic factors.

### Study setting and location

The location of the study is England. The data collected will only pertain to LAs in England and the populations of these LAs. There are a total of 314 lower tier LAs in England (April 2020 geography). Local authorities are responsible for food safety at the district and single tier level ^14^. The area of the LAs involved range from 5020 km ^2^ to 12 km ^2^ and populations range from 1,142,494 to 41,381 persons.

### Study overview and design

The study will be a longitudinal ecological study that uses data from 2010/11 to 2018/19. The data used will be secondary data and will be aggregated to LA level. The use of an ecological study design is pragmatic and will allow us to analyse the data at aggregated local authority level, over time each year.

### Study data

***EH expenditure***

Indicator(s):

➢ Total EH service expenditure in British sterling, per capita
  - Food safety expenditure as a proportion of total EH in British sterling, per capita

Local authority expenditure data is published by the Local Government Finance - Data Collection Analysis and Accountancy division of the Department for Communities and Local Government ^15^. This data describes the spending for Environmental and Regulatory services, this is also known as the revenue outturn data (RO5). The data we will use throughout the project are data that have been prepared by the Place Based Longitudinal Data Resource (PLDR) to provide consistent measures at the LA 2020 geography over time.

***EH services***

Indicator(s):

➢ Proportion of compliant establishments
➢ FTE per 100,000 of the population
➢ The percentage of interventions achieved
➢ The percentage of interventions achieved at unrated establishments
➢ The proportion of establishments subject to formal enforcement

The Consumer Data Research Centre (CDRC) provides information on the food hygiene rating result given to customer-facing food establishments participating on the Food Standard Agency’s (FSA) food hygiene rating scheme (FHRS). The data are held on behalf of local authorities. This data will be used to provide the food hygiene rating score which indicated the compliance level on a food establishment.

The FSA collate, analyse and publish data collected by LA food hygiene enforcement activities annually as local authority enforcement monitoring system (LAEMS) returns. This data will be used to derive EH indicators; the number of FTE positions, percentage of interventions achieved, the percentage of interventions achieved at unrated establishments, and proportion of enforcements.

***GI infection indicators***

Indicator(s):

➢ The number of hospitalisations with a diagnosis of acute gastrointestinal disease
➢ The number of NHS 111 calls due to diarrhoea and vomiting
➢ The number of specific GI pathogen detections for pathogens commonly associated with food poisoning:
  ○ *Salmonella* (Non-typhi)
  ○ *Campylobacter*
  ○ *Clostridium perfringens*
  ○ *E. coli *
  ○ *Listeria*

Hospital admissions due to GI infections are reported in hospital episode statistics (HES) which provide inpatient admissions for National Health Service (NHS) hospitals in England. The study will measure hospitalisations in England and will include cases defined using ICD-10 codes (A00–A09, K52.9) for all-cause acute gastroenteritis episodes (AGE).

The NHS telephone help line NHS 111, superseded by NHS Direct, allows people to access medical advice over the phone instead of in person consultations. The study will use data on the number of calls to NHS 111 reporting diarrhoea and vomiting symptoms in England.

The study will use laboratory pathogen data from Second Generation Surveillance System (SGSS) provided by the United Kingdom Health Security Agency (UKHSA), formerly Public Health England (PHE) which provide routinely collected national and regional surveillance data. The SGSS is the primary collection process by which data on positive cases of clinical significance and antimicrobial resistance (AMR) in England are recorded. Data requested from UKHSA SGSS will include *Salmonella non-typhi, Campylobacter, E. coli, Clostridium perfringens,* and *Listeria.*

The study will also request data from HPZone at UKHSA, a national system that monitors confirmed and suspected outbreaks of food poisoning, gastroenteritis, haemolytic uraemic syndrome and infectious bloody diarrhoea ^16^.

In addition, we will access data from EPINorth3, to provide detailed data on notifiable infections reported in the North East of England. The data will be linked to detailed information regarding exposures prior to infection by surveillance questionnaires and cover pathogens such as *Salmonella,* Shiga toxin producing *E. coli.*

### Community demographics

The measure of deprivation used will be the English Indices of Multiple Deprivation (IMD), which is produced by the UK Department for Communities and Local Government using local administrative data and data from the census ^17^. The IMD measure the relative levels of deprivation in England based on seven different bases of deprivation, these include: income deprivation, employment deprivation, education, skills and training deprivation, health deprivation and disability, crime, barriers to housing and services, and living environment deprivation ^18^. Population density data will be calculated using midyear population estimates and the area of LA’s in square kilometres provided by Office for National Statistics (ONS). As a proxy for accessibility to healthcare we explore the utility of data on distance from GP surgery.

We will use unemployment rate data, represented as the percentage of the economically active population aged 16 and above. In addition we will use gross domestic household income (GDHI), this measure details the amount of money that the population in the household sector have available to spend or save after income distribution measures. These data will be accessed from ONS and used to control for overall economic growth in each LA.

The data will cover years 2010/11 to 2018/19, and all data will be aggregated to the LA level. All outcome measures will be linked to a geocode which is then matched to the corresponding LA. Summary information on data, the source of the data and the corresponding measures can be found in Table 1.

**Table 1.** Data, source, corresponding measures.

Data	Source	Measure
**Local service expenditure**	Local Government Finance - Data Collection Analysis and Accountancy division of the Department for Communities and Local Government	Environmental health expenditure per capita (£)
**Environmental Health and** ** regulatory services**	Food Standards Agency: Local Authority Enforcement Monitoring System	Full time equivalent positions
		Interventions achieved at unrated
		Establishments subject to formal enforcement
		Interventions achieved
	Consumer Data Research Centre: Food Hygiene Rating Scheme	Compliance rating
**Gastrointestinal Infection**	Hospital Episode Statistics	Hospital admission for acute gastroenteritis
	National Health Service 111	Phone calls for diarrhoea and vomiting
	Second Generation Surveillance System	Laboratory confirmed cases of *Salmonella (Non-* *typhi), Campylobacter, Clostridium perfringens, * *E. coli, Listeria*
	HP Zone	Confirmed or suspected outbreaks of food poisoning, gastroenteritis, haemolytic uraemic syndrome, and infectious bloody diarrhoea
	EpiNorth3	Notifiable GI infection linked to survey data on exposure
**Community demographics**	Ministry of Housing, Communities & Local Government: Indices of Deprivation	Deprivation
	Office of National Statistics	Population Density
	Office of National Statistics	Gross Disposable Household Income
	Office of National Statistics	Unemployment

### Software

This project will use R studio version 4.3.0 for all data analysis.

### Ethical considerations

Ethical approval has been sought for this research; the University of Liverpool Ethics committee confirmed ethical approval will not be required. All data will be aggregated and anonymised therefore only data sharing agreements will be required.

### Data analysis

Once collected the data will be cleaned and aggregated to LA level, and variables will be derived. This will produce annual (repeated) local authority level measures. Exploratory and descriptive analysis will be carried out on the variables. Spatial mapping approaches will be used to visualise and explore geographically referenced data, to determine preliminary evidence supporting or contradicting *a priori* hypotheses. Missing data will be examined and dealt with where appropriate, using techniques such as imputation or elimination depending on the nature, extent, and reasons for missing data. Re sampling techniques may be used by incorporating a Bootstrap approach for multiple imputation purposes. Robustness of the variables will be assessed, as variables will be checked for collinearity and interdependency. Once the indicators to be carried forward for final analysis are determined, we will then carry out more formal statistical analysis as outlined below.

***Changes in spending (objective 1)***

Funding data will be analysed spatially and over time, to describe any changes. Annual expenditure per capita will be analysed between 2010/11 and 2018/19. Following a graphical assessment of trends in spending, a multivariable regression model incorporating random effects for LA’s and/or time as necessary will be fitted to quantify how changes in expenditure over time for LA’s association with explanatory variables such as deprivation, using IMD, and population density and LA type. This will allow us to begin exploration of how expenditure per capita varies in line with socioeconomic and demographic characteristics.

***Changes in EH indicators (objective 2)***

The EH indicators will be described spatially and overtime. Descriptive analysis will be carried out between the indicators and factors such as deprivation, and population density and food establishment density. We will also aim explore the level of tourism for each LA, as this may create additional pressure to local services of some areas.

***Association between changes in spending and changes in EH indicators (objective 3)***

To analyse the association between changes EH expenditure and trends in EH indicators we will use a fixed effects, methods which have been previously used in panel models for interrogating LA finance data ^19– 22^.

**Figure 1.**
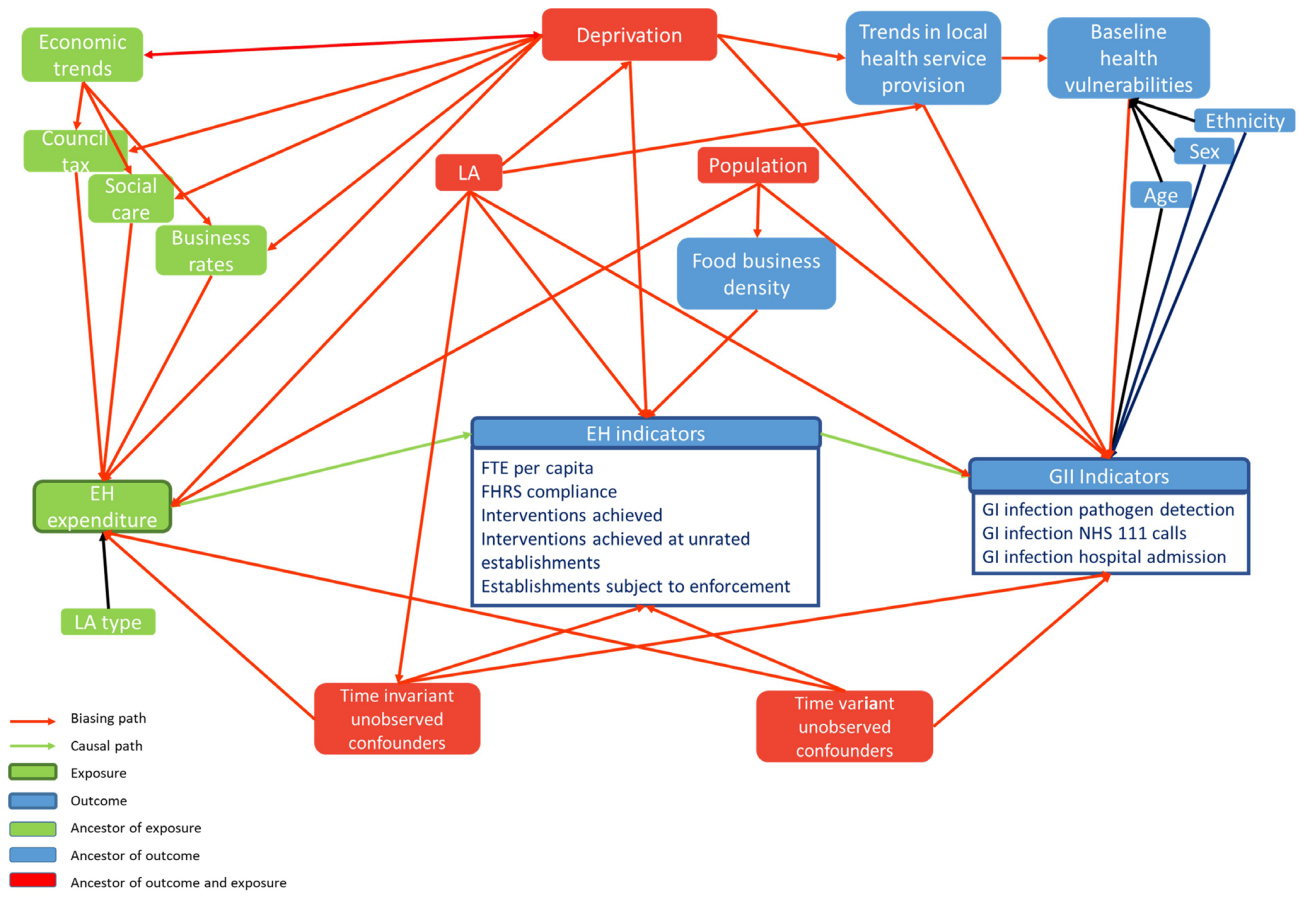
Diagram of causality between the exposure environmental health and regulatory service expenditure, and environmental health and regulatory service outcomes and gastrointestinal infection outcomes, also showing interactions with possible observed and unobserved confounders.

For the main analysis, we will use fixed effects panel regression, with fixed effects for LAs and individual years, to estimate the mean within-LA relationship between EH spend and EH indicators. We will account for secular trends across all LAs and observed and unobserved time invariant confounders (see Figure 1), including GDHI and unemployment. The fixed-effects approach removes unobserved confounders that vary between local authorities but are constant over time. We will log-transformed the exposure variable to account for expected diminishing returns on investment. Model results are, therefore, interpretable as change in EH indicators relative to the percentage change in spend. Initially we will use linear regression fixed effects models with exposure and outcome expressed as a rate per 100 population. In robustness tests we will use a fixed effects Poisson and linear regression model, including the log of the population as an offset.

***Changes in GI infection indicators (objective 4)***

The GI indicators will be described over time and spatially in the UK. This will allow the identification of times and areas where prevalence looks potentially high for further statistical investigation. Spatially referenced explanatory variables of interest include measures of deprivation and population density.

***Association between changes in EH services and changes in GI infection indicators (objective 5)***

We will develop spatiotemporal models to estimate the association between exposures and GI outcomes accounting for socioeconomic, demographic, and environmental factors. We will explore Poisson mixed model approach and explore the spatial clustering of residuals, such as using Moan’s I tests to examine if we need to incorporate a spatial model. As the outcomes are count data, we will utilise Poisson/negative binomial multivariable and multivariate regression models, depending on over-dispersion and zero inflation in the data. We will also evaluate the use of fixed effects for this objective, as this approach has been used to analyse LA spending association with health indicators ^19– 22^. The models will adjust for spatial autocorrelation if we detect its presence in the residuals by using ‘BYM’ models that extent the count regression modelling frameworks.

Methods will be developed, and a full data analysis plan will be established with further exploration and knowledge of the data.

### Power calculation

Power estimations are used in reference to sample size estimations, where they determine the number of subjects required to form the sample of the population, in aim to avoid type 2 error ^23^. The current study will not use sample data, rather it will use population level data. For this reason, a power calculation will not be required. However, sensitivity analysis and tests for robustness of our population data will be carried out to our assumptions.

### Timeline

The study will take place over 2 years, beginning in September 2022. Before the study begins, the relevant procedures regarding data sharing agreements and permissions will be actioned.

### Project governance

A stakeholder group will be established, consisting of EH workers from different LA’s who work with food hygiene. Initial meetings will be set up to introduce the project and gain relevant information. Throughout the project they will be able to contribute to the project. They will be updated periodically and be able to discuss and offer insights. or long-term secure storage of research data in a format.

The HES data to be used was made available to PLDR by NHS Digital under the data sharing agreement DARS-NIC-16656-D9B5T-v5.2 and has been risk -assessed by PLDR team and complies with HES small number analysis guidance. The data, as with all other data will be anonymised, non-identifiable and aggregated to the LA level. Safeguarded data will only be accessed, analysed, and stored on the university secure network, in line with management and practice guidelines.

### Dissemination and research findings

We will produce a report and policy brief of the key implications of our work to be shared with the stakeholder group and more widely to disseminate to UKHSA, public health professionals and policy makers. Findings should inform government and public health policy and strategy. The findings will be presented at professional and scientific conferences. The results will also be published in peer review open access publications.

## Strengths and limitations

A major strength is that we are able to utilise national representative data on environmental health and regulatory services expenditure, EH measures and GI infection outcomes. Importantly we can incorporate datasets that have been unanalysed in relation to funding cuts. 

GI infections are common, but most episodes do not result in a healthcare attendance. We therefore we intend to use a range of GI outcomes which cover a range of healthcare interactions, severities of illness and specificities. We will access HES admissions, laboratory confirmed cases of GI (specific pathogens) and NHS111 calls and online consultations for diarrhoea and vomiting. The use of multiple data sets will improve identification of any outliers from spatial and temporal trends.

Finally, a strength is the study design, the use of longitudinal data in a spatial- temporal approach is a main strength of this study. This approach provides insight to trends or changes in exposure and outcomes overtime, whilst accounting for variation geographically. This would be missed if we analysed data at a fixed point.

As this study is ecological in design it is susceptible to ecological fallacy; we can only infer the impact of funding cuts, not establish causation, particularly at the individual-level. However, the design is appropriate for evaluation of population and area level effects, as the exposure and primary outcomes (environmental health measures) are population level ones. Furthermore, we will attempt to ensure that the relationships derived from our area level data focus on the implications for areas and not people.

Like with all observational studies it is possible that there are residual confounders that will influence results that we are not able to account for. We will use stakeholder discussions to identify any further confounders that we could be adjusting for.

As with large data sets over long periods of time, missing data will likely be an issue, the methods will try to mitigate this via statistical methods mentioned.

There may be a limitation associated with the level and detail of some pathogen data due to changes in questionnaire requirements in the recent years. Due to changes in methodology in outbreak detection, there may be a reported increased detection of outbreaks due to improved techniques used. Further changes in data reporting and collection methods over the time period mentioned may change. We will attempt to identify changes through contact with stakeholders such as EHOs, FSA and UKHSA, so that changes may be described and where possible quantified to assist with suitable analytical adjustments. In addition, we will use a variety of relevant data sets for outcome indicators this will limit some of these issues by improving robustness.

Finally, EH expenditure accessed data does not provide the detail on what the money is spent on beyond service level, in each LA is likely to vary and in turn impact outcomes within and across LA’s both captured and uncaptured in this study. Furthermore, there will be differences across and within local authorities in regard to baseline health, in addition to healthcare provision.

## Discussion

This study will allow for a greater understanding of the impacts of funding reductions to local services, and how this may in turn impact GI infection health in England. The study will focus on EH services that provide public protection in forms of food hygiene and infectious disease control. The study aims to identify the effect of changes in expenditure to these services on EH services, and the change in GI infection outcomes. This study could provide a novel understanding of impacts of changes in funding on GI infection outcomes via the route of EH services and may provide a resource for policy makers to reference in the future.

## Data Availability

No data are associated with this article.
