# Supplementary material for: Understanding the impact funding cuts on Environmental and regulatory services and gastrointestinal infections: a longitudinal ecological study
Source: NIHR Open Res. 2024 Jan 5;3:41. Originally published 2023 Aug 14. [Version 2] doi: 10.3310/nihropenres.13426.2 (PMC11320035; doi:10.3310/nihropenres.13426.2)
Supplement: Peer review history

# Reviewer report: Reviewer response for version 2

**DOI:** 10.3310/nihropenres.14691.r31036

**Published:** 2024-02-15

**Authors:** Kun Li

**Affiliations:** 1 Nanjing Agricultural University, Nanjing,, China

**1) Abstract:** 

- Instead of using GI, use GIT because it will be more easy for readers to understand. Clearly describe what you mean by longitudinal ecological study (as just humans are involved in this study and longitudinal = time?), just for the reader's ease.
- Background is of sufficient quality.

**2) Introduction:**

- In line 2 of the introduction add detail of "GP". You can mark GP* and can give detail at the end of introduction or can highlight like Foodborn pathogens.
- English is of sufficient quality.
- Some words can be replaced with better synonyms like "disadvantaged" can be replaced with "deprived" or some other better synonym.

**3) Methodology:**

- In Figure 1, GI pathogens can be listed in brackets for quick and better understanding.
- Reason why only bacterial pathogens are being focussed and not Fungi, Amoeba, Giardia, Cryptosporidium and some Viral infection, also very common in GIT infections. Because in secondary data it is not difficult to get detail of different type of poisoning. In deprived communities these types of infestations/infection are also very common and responsible for the GI manifestations you mentioned in Table 1.

**4) Discussion:**

- Is of sufficient quality.

Overall, it seems good and is of sufficient quality.

Is the study design appropriate for the research question?

Yes

Is the rationale for, and objectives of, the study clearly described?

Yes

Are sufficient details of the methods provided to allow replication by others?

Yes

Are the datasets clearly presented in a useable and accessible format?

Yes

Reviewer Expertise:

Veterinary Medicine.

I confirm that I have read this submission and believe that I have an appropriate level of expertise to confirm that it is of an acceptable scientific standard.

# Reviewer report: Reviewer response for version 2

**DOI:** 10.3310/nihropenres.14691.r31035

**Published:** 2024-02-15

**Authors:** Paolo Lauriola

**Affiliations:** 1 International Society of Doctors for Environment, Geneva, Switzerland

In my opinion, the article in its present form is acceptable for indexing, and I do not have any further comments to add.

Is the study design appropriate for the research question?

Partly

Is the rationale for, and objectives of, the study clearly described?

Partly

Are sufficient details of the methods provided to allow replication by others?

No

Are the datasets clearly presented in a useable and accessible format?

Not applicable

Reviewer Expertise:

Environmental Epidemiology, Public Health

I confirm that I have read this submission and believe that I have an appropriate level of expertise to confirm that it is of an acceptable scientific standard.

# Reviewer report: Reviewer response for version 1

**DOI:** 10.3310/nihropenres.14561.r30515

**Published:** 2023-10-09

**Authors:** Paolo Lauriola

**Affiliations:** 1 International Society of Doctors for Environment, Geneva, Switzerland

**In general**

- The overall objective of the study is important. As the Authors are presenting a proposal, I feel that it would be underlined that this article is presented to collect comments and suggestions, which are welcome.
- The overall logical framework should be revised
- There are too many Acronyms which are misleading

**In particular**

**Abstract/Introduction:**

- I think the use of the acronym (EH) is improper. Environmental Health has a very general meaning, whilst, throughout the article, it refers to the services/indicators dealing with environmental and food controls. It also used the acronym EHO, the regulatory service responsible for food safety and infectious disease control to some extent. I suggest reducing the number of abbreviations and giving them a precise meaning. Even considering the specificity of the UK NHS organisation, it must be remembered the article will be read by overseas people, too.

**Plain language summary: **is it necessary? Maybe it would be used to introduce the study to lay people, but this is a scientific article. I’d delete it

**Introduction: **

- Please explain what the IID2 survey is
- I would move the sentence “Increased expenditure on food safety and sanitation services have shown to be associated with reductions in notifiable enteric disease” after “Though sociodemographic and spatial inequalities of GI infection are well described, the drivers behind them remain to be understood”. The expenditure on food safety is one of the determinants of the spread of IID, described before it is currently placed.

**Methods:**

- Arrows/dashes, please check
- The study overview and design must described in more detail
- Please explain what NHS
- Why don’t you use GPs’ data? UKHSA has a very effective Syndromic surveillance system ( https://assets.publishing.service.gov.uk/government/uploads/system/uploads/attachment_data/file/1136854/UKHSA_GPinHoursEngBulletin2023Wk06.pdf)
- Fig. 1 should be presented as the study hypothesis to be investigated. Instead, it seems a result of a study which hasn’t been carried out yet. Please change and simplify

Is the study design appropriate for the research question?

Partly

Is the rationale for, and objectives of, the study clearly described?

Partly

Are sufficient details of the methods provided to allow replication by others?

No

Are the datasets clearly presented in a useable and accessible format?

Not applicable

Reviewer Expertise:

Environmental Epidemiology, Public Health

I confirm that I have read this submission and believe that I have an appropriate level of expertise to confirm that it is of an acceptable scientific standard, however I have significant reservations, as outlined above.

# Response

**Published:** 2023-12-22

**Authors:** Lauren Murrell

**Affiliations:** IVES, University of Liverpool, Liverpool, England, UK

I think the use of the acronym (EH) is improper. Environmental Health has a very general meaning, whilst, throughout the article, it refers to the services/indicators dealing with environmental and food controls. It also used the acronym EHO, the regulatory service responsible for food safety and infectious disease control to some extent. I suggest reducing the number of abbreviations and giving them a precise meaning. Even considering the specificity of the UK NHS organisation, it must be remembered the article will be read by overseas people, too.

 Response: Thank you, EH refers to the services Environmental and regulatory services, sometimes environmental health and regulatory services is used and sometimes the term regulatory services is used. However, I think it can be improved and that environmental and regulatory services is a more specific term. I will refer to EH as ER – environmental and regulatory services. We have therefore changed each occurrence of environmental health and regulatory services / EH to environmental and regulatory services / ER.

**Plain language summary: **is it necessary? Maybe it would be used to introduce the study to lay people, but this is a scientific article. I’d delete it

 Response: Thank you for your comment, this section is a requirement by the journal NIHR open. The lay summary has been generated through our Patient Public involvement and engagement panel and our lay co-author.

Please explain what the IID2 survey is

 Response: Thank you for this suggestion, I have amended this and added detail of the IID2 study on page 4 line 20, it is a brief description, but it should give enough detail to the reader. The sentence now reads as

  “Cross sectional analysis using UK population data from the IID2 survey, a study which describes IID in the community across the UK, identified increased symptom severity amongst those of more disadvantaged socioeconomic…”

I would move the sentence “Increased expenditure on food safety and sanitation services have shown to be associated with reductions in notifiable enteric disease” after “Though sociodemographic and spatial inequalities of GI infection are well described, the drivers behind them remain to be understood”. The expenditure on food safety is one of the determinants of the spread of IID, described before it is currently placed.

 Response: Thank you for this suggestion. We believe that whilst this would be a possible, it would make more sense to leave this sentence where it is as it is an important part of the paragraph it introduces .

The study overview and design must described in more detail

 Response: Thank you for your suggestions, I have added some more detail to the overview and design see page 6; line 30-38, page 7; lines 1-2

Please explain what NHS

 Response: Thank you, we have included additional brief detail on what the NHS is on page 4 lines 9-10. The sentence now reads as:

  “…resulting in an estimated annual cost of £60m to the National Health Service (NHS) ^2^ , the government-funded health service for the UK…”

Why don’t you use GPs’ data? UKHSA has a very effective Syndromic surveillance system ( https://assets.publishing.service.gov.uk/government/uploads/system/uploads/attachment_data/file/1136854/UKHSA_GPinHoursEngBulletin2023Wk06.pdf)

 Response: Thank you for your suggestion, unfortunately this would not be suitable for this study due to the geographic coverage of GP data, it is not available at the LA level which is required for this study. However, the UKHSA uses NHS 111 for syndromic surveillance, which we will be using alongside HP zone outbreak reports which we can access at the required level.

Fig. 1 should be presented as the study hypothesis to be investigated. Instead, it seems a result of a study which hasn’t been carried out yet. Please change and simplify

Response: Correct, the figure is not intended to be presented as the results.

 This Directed acyclic graph (DAGs) has been generated a-priori based on prior professional knowledge, subject expertise and the literature. It provides a simple a way for us to present the knowledge, theories and assumptions about causal relationships between variables prior to study data collection and analysis. We have therefore moved the DAG to the study overview and design section and added further explanation in the text, starting on page 6 line 36:“We have constructed a Directed Acyclic Graph (DAG) to clarify the study aims and objectives (Figure 1). The DAG allows us conceptualise theories, assumptions and bias in causal pathways between exposures and outcomes, which relate to the study objectives. Additionally, we specify a-priori the minimal set of covariates required for study in the DAG and in the next section of the protocol"

Other notes for reviewer

 Since the submission of the protocol, it was decided that in addition to food safety we would also assess the spending line - animal and public health infectious disease control . This is due to the responsibilities of this sub stream in infectious disease control, therefore analysing this in addition to food safety expenditure will provide a more comprehensive understanding of crucial services within Environmental and regulatory. We include it as an indicator on page 7 line 14 

 “Animal and Public Health; Infectious Disease Control expenditure as a proportion of total ER in British sterling per capita”

# Reviewer report: Reviewer response for version 1

**DOI:** 10.3310/nihropenres.14561.r30410

**Published:** 2023-10-04

**Authors:** Kun Li

**Affiliations:** 1 Nanjing Agricultural University, Nanjing,, China

The title of the article **“** ******Understanding the impact of local funding cuts on environmental health and regulatory services and gastrointestinal infection outcomes: a longitudinal ecological study protocol****** **”** is not capturing the attention of reader somewhat, seems not interesting, not engaging the reader. It may be simple, concise and interesting.

**Introduction:** The choice of words should be simple somewhere in the introduction and so on. However, ****sufficient background information is available in the introduction and it justify the study.

**In plain language summary:** The reason to mention stomach bug especially should be clearly mentioned.

**In methodology:** The population range in the section “Study setting and location” is greater, about 1,101,113. This may be lower. ****Also, the ****authenticity and validity of the data can be rechecked, as it is not a primary data, however ensuring the strength of data validity as discussed in the given article will eliminate the outliers, hopefully. ****The reason to not use the *Salmonella typhi* as an indicator of GIT health may be mentioned. Alterations in the gut microbiota diversity may also be included for detailed information regarding GIT disturbances. During the process of data collection, interpretation and dissemination of information the one health professionals from all sectors should participate to improve the accuracy of data.

**Discussion:** Decrease in funding sources increases the stress level in the population due to unavailability of different services, which leads to GIT disturbances and serious health outcomes. This type of survey may also be included in the given study plan to support the results.

 In the result section, manifestation of the data in the form of tables consisting of the exact secondary data may improve the visualization and will attract the reader attention

 Overall, the different sections of this article need minor improvements and overall it seems good.

 The title of the article **“** ******Understanding the impact of local funding cuts on environmental health and regulatory services and gastrointestinal infection outcomes: a longitudinal ecological study protocol****** **”** is not capturing the attention of reader somewhat, seems not interesting, not engaging the reader. It may be simple, concise and interesting.

**Introduction:** The choice of words should be simple somewhere in the introduction and so on. However, ****sufficient background information is available in the introduction and it justify the study.

**In plain language summary:** The reason to mention stomach bug especially should be clearly mentioned.

**In methodology:** The population range in the section “Study setting and location” is greater, about 1,101,113. This may be lower. ****Also, the ****authenticity and validity of the data can be rechecked, as it is not a primary data, however ensuring the strength of data validity as discussed in the given article will eliminate the outliers, hopefully. ****The reason to not use the *Salmonella typhi* as an indicator of GIT health may be mentioned. Alterations in the gut microbiota diversity may also be included for detailed information regarding GIT disturbances. During the process of data collection, interpretation and dissemination of information the one health professionals from all sectors should participate to improve the accuracy of data.

**Discussion:** Decrease in funding sources increases the stress level in the population due to unavailability of different services, which leads to GIT disturbances and serious health outcomes. This type of survey may also be included in the given study plan to support the results.

 In the result section, manifestation of the data in the form of tables consisting of the exact secondary data may improve the visualization and will attract the reader attention

 Overall, the different sections of this article need minor improvements and overall it seems good.

Is the study design appropriate for the research question?

Yes

Is the rationale for, and objectives of, the study clearly described?

Yes

Are sufficient details of the methods provided to allow replication by others?

Yes

Are the datasets clearly presented in a useable and accessible format?

Yes

Reviewer Expertise:

Veterinary Medicine.

I confirm that I have read this submission and believe that I have an appropriate level of expertise to confirm that it is of an acceptable scientific standard, however I have significant reservations, as outlined above.

# Response

**Published:** 2024-01-02

**Authors:** Lauren Murrell

**Affiliations:** IVES, University of Liverpool, Liverpool, England, UK

The title of the article **“ *Understanding the impact of local funding cuts on environmental health and regulatory services and gastrointestinal infection outcomes: a longitudinal ecological study protocol*”** is not capturing the attention of reader somewhat, seems not interesting, not engaging the reader. It may be simple, concise and interesting.

 Response: Thank you for this feedback, whilst this is a lengthy title, it is important that the title gives a clear and accurate description of the study. But we have removed redundant words to shorten this title and make it more concise. The title will now read:

**“ *Understanding the impact of funding cuts on Environmental and regulatory services and gastrointestinal infections: a longitudinal ecological study*”** 

**In plain language summary: **The reason to mention stomach bug especially should be clearly mentioned.

 Response: The lay summary has been generated through our Patient Public involvement and engagement panel and our lay co-author. They are satisfied with how stomach bugs (GI) are discussed

**In methodology: **The population range in the section “Study setting and location” is greater, about 1,101,113. This may be lower. ****Also, the ****authenticity and validity of the data can be rechecked, as it is not a primary data, however ensuring the strength of data validity as discussed in the given article will eliminate the outliers, hopefully.****

 Response: The population estimate refers to the 2020 estimate provided by the Office of National Statistics; the source data used in each article will be specified. While other sources and years may provide different population estimates to my example, I will keep this estimate as it is the most recent estimate that is relevant to my study period, additionally ONS is the most reliable source to use.

The reason to not use the *Salmonella typhi* as an indicator of GIT health may be mentioned.

 Response:  

 We have decided not to use Salmonella typhi as it is not endemic in the England/UK and is most commonly travel related, therefore it is not included as a pathogen of interest for this work. Including it could lead to inaccurate conclusions in regards to our question. We have also added the following information to the GI infections indicators:

 “These pathogens have been selected through an expert group of health professionals affiliated to the NIHR Health Protection Research Unit in GI.”

Alterations in the gut microbiota diversity may also be included for detailed information regarding GIT disturbances.

 Response:  Thank you for this suggestion, this would be an interesting area of research, our study however will focus on health inequalities in infectious food borne disease, this would be outside of our inclusion criteria for this study

During the process of data collection, interpretation and dissemination of information the one health professionals from all sectors should participate to improve the accuracy of data.

 Response:  Thank you for this suggestion. We have and will continue communicate with professionals from UK Heath Security Agency and, local authorities, including environmental health staff, epidemiologists, and microbiologists. This gives us extra insight to these data and how it may be used and interpreted.

**Discussion: **Decrease in funding sources increases the stress level in the population due to unavailability of different services, which leads to GIT disturbances and serious health outcomes. This type of survey may also be included in the given study plan to support the results.

 Response: This is a very interesting suggestion but there is no capacity or resource for primary data collection in this study and this does not sit within out study objectives.

In the result section, manifestation of the data in the form of tables consisting of the exact secondary data may improve the visualization and will attract the reader attention

Overall, the different sections of this article need minor improvements and overall it seems good.

 Response: Thank you for this suggestion, but we will not be including data in this article as it is a study protocol and as such, does not include results the priority of this paper is to present the plan for the data and the study approach. I will however be presenting the exact data and results in open access publications.

Other notes for reviewer

 Since the submission of the protocol, it was decided that in addition to food safety we would also assess the spending line - animal and public health infectious disease control. This is due to the responsibilities of this sub stream in infectious disease control, therefore analysing this in addition to food safety expenditure will provide a more comprehensive understanding of crucial services within Environmental and regulatory. We include it as an indicator on page 7 line 14 

 “Animal and Public Health; Infectious Disease Control expenditure as a proportion of total ER in British sterling per capita”
